# Temporal Stability, Reproducibility and Predictability of Whole-Body Sweat Sodium Concentration During Prolonged Cycling in the Heat with *Ad Libitum* and Programmed Drinking

**DOI:** 10.3390/nu18060989

**Published:** 2026-03-20

**Authors:** Eric D. B. Goulet, David Jeker, Pascale Claveau, Thomas A. Deshayes, Timothée Pancrate, Mohamed El Fethi Abed, Antoine Jolicoeur Desroches, Martin D. Hoffman, Philippe Gendron, Claude Lajoie, Lisa Lehmann

**Affiliations:** 1 Faculty of Physical Activity Sciences, University of Sherbrooke, Sherbrooke, QC J1K 2R1, Canada; coach.jeker@gmail.com (D.J.); pascale.claveau2@usherbrooke.ca (P.C.); thomas.deshayes@usherbrooke.ca (T.A.D.); timothee.pancrate@usherbrooke.ca (T.P.); mohamed.el.fethi.abed@usherbrooke.ca (M.E.F.A.); antoine.jolicoeur-desroches@usherbrooke.ca (A.J.D.); 2 Performance, Hydration and Thermoregulation (PHT) Laboratory, University of Sherbrooke, Sherbrooke, QC J1K 2R1, Canada; 3 Research Center on Aging, University of Sherbrooke, Sherbrooke, QC J1H 4C4, Canada; 4 Independent Researcher, El Dorado Hills, CA 95762, USA; md.hoffmanmd@gmail.com; 5 Department of Physical Activity Sciences, Université du Québec à Trois-Rivières, Trois-Rivières, QC G8Z 4M3, Canada; philippe.gendron@uqtr.ca (P.G.); claude.lajoie@uqtr.ca (C.L.); 6 Independent Researcher, 06700 Nantes, France; lisa.lehmann@univ-nantes.fr

**Keywords:** exercise, replacement, sodium, sweat

## Abstract

**Background:** Leading sports medicine and nutrition organizations recommend replacing sodium losses during prolonged exercise; however, practical guidance for implementing sodium replacement strategies remains limited. Estimating sodium needs during exercise requires assessment of both whole-body sweat sodium concentration (WBSSC) and sweat rate. **Objectives:** This study focused on WBSSC by examining its temporal stability, reproducibility, and predictability during prolonged cycling exercise while drinking according to two hydration strategies. **Methods**: Using a randomized, crossover, counterbalanced design, eight highly trained men completed two 5 h cycling sessions (183 ± 14 W, 30 °C) while consuming fluids either in a programmed (P) or *ad libitum* (A_L_) fashion. Sweat was collected with patches applied on the forearm for ~20 min before sampling, which occurred at ~40, 130, 220, and 290 min. Local sweat sodium concentration was converted to WBSSC using a validated equation. **Results**: A main effect of time was observed for WBSSC (*p* < 0.05), with only the 40 min time point differing from later measurements; no condition or interaction effects were detected. The within-trial typical variation in WBSSC was 7.2 mmol·L^−1^ for P and 6.1 mmol·L^−1^ for A_L_, while the between-trial typical variation was 5.6 mmol·L^−1^. The WBSSC measured at 40 min predicted mean exercise WBSSC with good precision and moderate stability (y = 0.2738 + 1.3397x, R^2^ = 0.87, standard error of the estimate = 5.4 mmol·L^−1^, 95% confidence interval slope = 0.82–1.86 mmol·L^−1^). **Conclusions**: These findings indicate that during prolonged cycling exercise, WBSSC (1) varies trivially within and between trials; (2) can reasonably be predicted using a single sweat sample and; (3) is not influenced by P or A_L_ drinking.

## 1. Introduction

Sweat production and evaporation from the skin surface are essential for dissipating metabolic heat during exercise [1]. Although sweat is composed primarily of water, it also contains several electrolytes, of which sodium is the most abundant and physiologically relevant [2]. When high sweat rates are sustained for prolonged periods, substantial sodium losses may occur [1]. Accordingly, multiple position statements by leading organizations recommend sodium replacement during prolonged exercise when sweat losses are substantial [3,4,5]. However, these organizations provide little guidance regarding the practical implementation of sodium replacement strategies during exercise.

The lack of clear guidance likely reflects the scarcity of controlled-laboratory research addressing sodium replacement during prolonged exercise [6,7,8]. To begin addressing this gap, McCubbin [6] recently published a modeling study estimating sodium replacement requirements during exercise while accounting for exercise duration, body mass, whole-body sweat rate, whole-body sweat sodium concentration (WBSSC), baseline plasma sodium concentration, potassium balance, and fluid replacement rate. Based on this model, which is likely not universally applicable to all prolonged exercise scenarios, sodium replacement guided by sweat composition testing appears most relevant when exercise duration exceeds 4 h, sweat rate is less than 1800 mL·h^−1^, fluid replacement exceeds 70% of whole-body sweat losses, and WBSSC is at least 40 mmol·L^−1^. When these criteria are met, McCubbin [6] proposed sodium replacement targets based on the proportion of the whole-body sweat losses replaced by fluid intake and the athlete’s WBSSC.

Although McCubbin’s paper [6] provides relevant and tailored guidance regarding the amount of sodium that should be replaced during prolonged exercise, it does not resolve several practical issues that are needed by field practitioners to emit recommendations with confidence. First, the temporal behavior of WBSSC during prolonged exercise has been incompletely characterized. It has been demonstrated that WBSSC increases over time during bouts of exercise varying in duration from 90 min to 3 h [9,10,11]. On the other hand, Montain et al. [12] reported minimal changes in WBSSC across three measurements obtained during 7 h of exercise, but their protocol involved intermittent, low-intensity exercise in participants of unspecified training status, with sweat samples collected over 1 h periods, which may have confounded WBSSC estimates [1]. Second, the reproducibility of WBSSC between prolonged bouts of exercise has not been directly determined. Finally, the extent to which WBSSC can be accurately predicted from sweat composition testing, and whether the drinking strategy impacts WBSSC, is unknown.

In 2021, our laboratory published a controlled laboratory study examining the influence of programmed (P) and *ad libitum* (A_L_) drinking during a 5 h fixed-intensity cycling exercise followed by a 20 km time trial [13]. Fluid replacement exceeded 80% of whole-body sweat losses in both trials. In that paper, we reported the mean WBSSC observed in each condition, which were not significantly different. However, as secondary outcomes, we took serial measurements of forearm sweat sodium concentration throughout exercise, but these data have not previously been reported. The present study used these measurements to address the unresolved questions outlined above. Therefore, the purpose of the present study was to determine during two 5 h laboratory-controlled cycling sessions performed under two different drinking strategies, i.e., P and A_L_, (1) the temporal behavior of WBSSC within each trial; (2) the reproducibility of WBSSC between trials and; (3) the ability of a single sweat sample collected 40 min after exercise onset to predict the mean WBSSC observed during prolonged exercise.

## 2. Materials and Methods

### 2.1. Participants

Participation was opened to both men and women; only men, a total of 8, agreed to participate. All were highly trained endurance cyclists and triathletes. Baseline physical characteristics of all participants are detailed in Table 1. After a comprehensive explanation of the study goals, procedures, and risks, and after addressing any questions, participants provided both oral and written consent. The study received approval from the CIUSSS Estrie-CHUS Ethics Committee (#2019-2837).

### 2.2. Overview of the Experiments

Participants completed two experimental trials consisting of 5 h of fixed-intensity cycling, followed by a 20 km time trial. An assessment of muscle cramping susceptibility was additionally performed following the time trial. During exercise, participants consumed fluid according to two different hydration strategies. Results related to the impact of hydration strategies on body water and sodium balance, subjective perceptions, cardiovascular and thermoregulatory functions, endurance performance, and muscle cramping have been previously published [13]. Prior to the experiments, participants completed a preliminary visit and a familiarization trial for baseline measurements. Experiments were conducted in a randomized, crossover, and counterbalanced fashion, at the same time of day, and were separated by 14 to 28 days.

### 2.3. Preliminary Visit and Familiarization Trial

Upon arrival at the laboratory, participants first voided, after which post-void nude body mass and height were measured using a digital scale (±20 g, BX-300+, Atron Systems, West Caldwell, NJ, USA) and a wall-mounted stadiometer, respectively. Body composition was assessed using dual-energy X-ray absorptiometry (Lunar Prodigy, GE Healthcare, Chicago, IL, USA). Peak oxygen consumption (V̇O_2peak_) was measured while participants used their own bicycles mounted on a Computrainer^TM^ (Racermate, Seattle, WA, USA). The validity and reliability of the Computrainer^TM^ used in the study have been previously determined [14]. Gas exchange was analyzed using a gas analysis system (Cosmed Quark CPET, Cosmed, Chicago, IL, USA), calibrated with gases of known concentration. Participants completed a familiarization trial to confirm the workloads to be used for the fixed-intensity cycling periods, estimate whole-body sweat rates, and determine the water intake rates required during P to maintain body mass losses at ~1%.

### 2.4. Pre-Experimental Procedures

Participants were instructed to record their food and fluid intake, including time and quantity, during the 48 h preceding the familiarization trial, and to replicate these diets before the subsequent experimental trials. For the 24, 48, and 72 h prior to the familiarization and experimental trials, participants were required to maintain the same training routines or rest, avoid sports supplements, and cease lower limb strength training, respectively. One hour before bedtime on the night preceding the familiarization and experimental trials, as well as one hour before these visits, participants were required to drink 250 mL of water. After consuming the prescribed fluid on the trial day, participants abstained from further food and fluid intake. Finally, participants were instructed to wear the same cycling clothing throughout the entire exercise period in each experimental trial.

### 2.5. Experimental Protocol

#### 2.5.1. Arrival at Laboratory

Upon arrival, participants’ cycling clothing and shoes were weighed. The mid-dorsal region of the forearm, where absorbent patches were applied to collect sweat, was then cleaned with 70% alcohol swabs, shaved with a disposable razor, and lightly abraded using 3M abrasive paper to remove dead skin cells and maximize patch adhesion. Participants then voided their bladder and collected a midstream urine sample for urine specific gravity and urine osmolality assessment. Participants were subsequently weighed to assess baseline body mass.

#### 2.5.2. Experiments

After entering the environmental chamber, participants rested on their bicycles for 10 min, perceived thirst was measured and a capillary blood sample was collected via a finger prick (BD Microtainer, Mississauga, ON, Canada). Participants then completed 5 h of cycling on the Computrainer^TM^ at a fixed power output corresponding to 61% of V̇O_2peak_. The Computrainer^TM^ was calibrated according to the manufacturer recommendation prior to each trial. Exercise was performed at 30 °C and 35% relative humidity, with 500–600 W·m^−2^ of radiance. Three large fans positioned in front of the bicycles provided a whole-body airflow at approximately 25–30 km·h^−1^. During exercise, participants consumed water either A_L_ or in fixed volumes every 15 min to limit body mass loss at ~1% (P). Oxygen consumption was measured for 5 min at 20, 60, 120, 180, 240, and 290 min of the cycling periods. Participants consumed sodium-containing energy gels (GU Roctane, GU Energy Labs, Berkeley, CA, USA) at fixed, individualized times during exercise. Carbohydrate intake (total of 313 ± 25 g) and sodium intake (total of 2133 ± 157 mg) were maintained constant between experimental trials. Immediately following the 5 h exercise periods, participants urinated, dried off, and were weighed.

#### 2.5.3. Measurements

Thirst was measured with an arbitrary (1–11) scale [15]. Urine and whole blood osmolality were assessed using the freezing point depression technique (Micro Osmometer, Osmette, Precision Systems Inc., Natick, MA, USA). Whole-body sweat losses were computed while uncorrected and corrected for respiratory water losses and metabolic mass losses, as described by Mitchell et al. [16].

Local sweat production was collected using self-adhesive absorbent patches (Tegaderm + Pad, 6 × 10 cm, 2.5 × 6 cm absorbent pad, 3M, USA) [17]. The skin was thoroughly cleaned with deionized water and dried with sterile gauze before each patch application. Patches were firmly pressed onto the skin for approximately 5 sec to ensure an optimal seal and prevent sweat leakage. Patches were removed using sterile tweezers before becoming saturated, as determined by visual inspection. Patches were applied on the skin for ~20 min at min 20, 110, 200 and 270. They were therefore removed at ~min 40, 130, 220 and 290. Following removal, patches were immediately placed into zip-top resealable plastic bags. At the end of the trials, patches were transferred to 15 mL tubes and centrifuged at room temperature for 10 min at 3500 rpm to extract sweat. Extracted sweat was transferred into 2 mL microcentrifuge tubes and stored at −20 °C until analysis. Sweat sodium concentration was analyzed using the ion-selective electrode technique (B-722 Laqua Twin, Horiba Scientific, Edison, NJ, USA) [17]. Measurement and calibration procedures have been described in detail elsewhere [18]. Local sweat sodium concentration values were converted to WBSSC using the regression equation reported by Baker et al. [19] for the posterior midforearm. At present, this equation represents the only available approach for performing this conversion.

### 2.6. Statistical Analyses

#### 2.6.1. Hydration Status Prior to the Experimental Trials

Paired *t*-tests were used to compare differences in body mass, urine specific gravity and thirst between trials. One result was missing for whole-blood osmolality. Hence, for this variable, a linear mixed-effects model (fixed effects: conditions; random effects: participants) was used to compare results between trials. Normality of residuals (differences for paired *t*-tests) was assessed using the Shapiro–Wilk test and visual inspection of Q-Q plots. Statistical significance was set at *p* ≤ 0.05. Values are reported as means ± SD. Analyses were conducted using IBM SPSS Statistics (version 21, New York, NY, USA).

#### 2.6.2. Temporal Behavior of WBSSC

Linear mixed-effects models (fixed effects: time and conditions; random effects: participants) were used to determine whether differences in WBSSC within or between trials were statistically significant. If necessary, post hoc analyses were performed using the false discovery rate procedure [20]. Normality of residuals was assessed using the Shapiro–Wilk test and visual inspection of Q-Q plots. Statistical significance was set at *p* ≤ 0.05. Values are reported as means ± SD. There were two missing values for WBSSC (one in each condition). Analyses were conducted using IBM SPSS Statistics (version 21, New York, NY, USA).

#### 2.6.3. Reproducibility of WBSSC

Variations in WBSSC within and between trials were assessed using the change in measurement, the typical variation in measurement (within-subject standard deviation), and the coefficient of variation [21,22]. The change in measurement represents the difference between two time points. The typical variation in measurement reflects the inherent variability associated with repeated assessments of the same variable within individuals, and the coefficient of variation represents this variability expressed as a percentage of the mean. For pairwise comparisons, the typical variation in measurement was calculated as the standard deviation divided by √2 [22], whereas for analyses involving more than two comparisons, it was estimated as the square root of the residual covariance obtained from a linear mixed-effects model [23]. Paired *t*-tests were used to determine whether differences in WBSSC between two time points were systematic [21]. Intraclass correlation coefficients (absolute agreement, two-way mixed effects, single measurement) were computed for each between-trial measurement time points. Analyses were conducted using Microsoft Excel (version 2502, Redmond, WA, USA) and IBM SPSS Statistics (version 21, New York, NY, USA).

#### 2.6.4. Prediction of WBSSC

A regression analysis was conducted to determine whether the 40 min sweat sample can be used to adequately predict the time-weighted average WBSSC of the entire exercise bout. The time-weighted WBSSC for each individual was computed with the following equation:

(90 * min/250 ^#^ min·WBSSC of time point 130) + (90 ** min/250 min·WBSSC of time  point 220) + (70 *** min/250 min·WBSSC of time point 290),

where

* is the elapsed time between min 40 and 130;
** is the elapsed time between min 130 and 220;
*** is the elapsed time between min 220 and 290 and;
^#^ is the elapsed time between min 40 and 290.

Normality of residuals was assessed using the Shapiro–Wilk test and visual inspection of Q-Q plots. Statistical significance was set at *p* ≤ 0.05. Heteroscedasticity was evaluated by plotting the residuals against the predicted values. Autocorrelation was evaluated using the Durbin–Watson test. Regression model stability was evaluated using complementary approaches assessing both the influence of individual observations and the sampling variability of model parameters. Influence diagnostics were performed using Cook’s distance to identify observations exerting disproportionate effects on regression estimates. In addition, model stability was examined through bootstrap resampling procedures (10,000 iteration), whereby the regression model was repeatedly refitted on resampled datasets to quantify the variability of regression coefficients across repeated samples. Bootstrap resampling provides an empirical estimate of sampling variability and is recommended for evaluating the stability and reproducibility of regression models [24]. Parameter stability was quantified as the relative variability of the regression coefficient, calculated as the ratio between the bootstrap standard deviation and the coefficient estimate. Relative variability values < 10%, 10–20%, and >20% were interpreted as indicating high, moderate, and limited stability, respectively [25]. Analyses were conducted using IBM SPSS Statistics (version 21, New York, NY, USA).

#### 2.6.5. Statistical Power

A posteriori statistical power analysis demonstrated that the power to detect differences in WBSSC across time within trials was 0.88. Power calculations were based on a sample size of 8 participants, an effect size (f) of 0.5 [10], an α level of 0.05, four repeated measurements, and a correlation coefficient of 0.5. Analyses were conducted using G*Power (v. 3.1.9.7).

## 3. Results

### 3.1. Hydration Status Prior to the Experimental Trials

Indices of hydration status prior to the experimental trials are presented in Table 2. These data indicate that participants began both trials well hydrated, in a similar hydration state, and with the kidneys in a comparable state of water conservation, as indicated by the urine specific gravity values.

### 3.2. Cycling Trial Duration and Intensity

As per the study design, participants cycled for 5 h at the same power output (183 ± 14 W) in both trials. Mean V̇O_2_ during exercise was 2.9 ± 0.2 L·min^−1^ with P and 2.9 ± 0.3 L·min^−1^ with A_L_ (*p* = 0.45), corresponding to a mean exercise intensity of 61 ± 4% of V̇O_2peak_ (P: 62 ± 3%; A_L_: 61 ± 5%, *p* = 0.44).

### 3.3. Fluid and Electrolyte Balance

Body mass loss, total water intake, total urine production, metabolic mass loss, metabolic water production, respiratory water loss, whole-body sweat loss, whole-body sweat rate, body water balance, total sodium intake, total whole-body sodium loss, and sodium balance data are reported elsewhere [13]. The percentage of body mass loss at the end of exercise was 0.9 ± 1.2% with P and 1.9 ± 0.9% with A_L_ (*p* > 0.05). The percentage of whole-body sweat losses replaced by water intake was 123 ± 27% for P and 96 ± 31% for A_L_ (*p* = 0.06). Whole-body sweat losses uncorrected for metabolic mass losses and respiratory water losses were 6.04 ± 0.70 L for P and 6.08 ± 0.57 L for A_L_ (*p* = 0.74), corresponding to whole-body sweat rates of 1.22 ± 0.11 L·h^−1^ and 1.20 ± 0.14 L·h^−1^, respectively (*p* = 0.74). The percentage of whole-body sweat losses replaced by fluid intake when these corrections were omitted was 106 ± 23% for P and 83 ± 24% for A_L_ (*p* = 0.07).

### 3.4. Changes in Whole-Body Sweat Sodium Concentration Within and Between Trials

**Figure 1:**
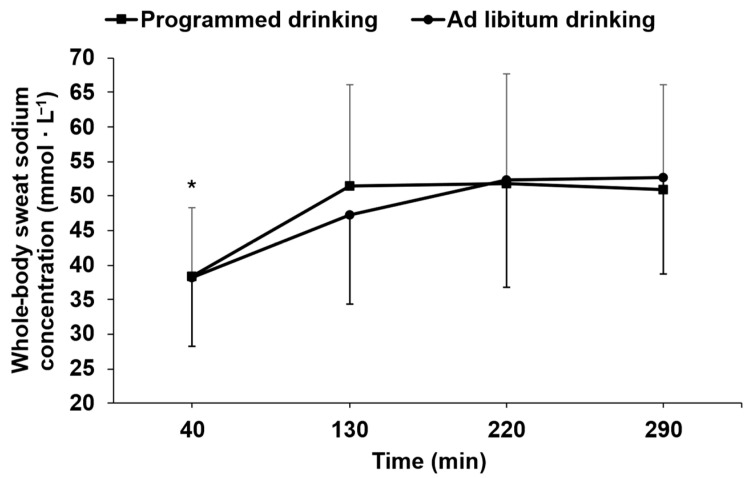
Temporal changes in whole-body sweat sodium concentration for both exercise trials. * = Time effect (*p* < 0.05), significantly different from min 130, 220 and 290. Values presented at each time point are representative of a sweat collection that occurred for 20 min leading up to that time point. Values are reported as mean ± SD.

Figure 1 illustrates the temporal changes in WBSSC during both exercise trials. A main effect of time was observed (*p* < 0.05), whereas no effect of condition or time × trial was detected (*p* > 0.05). Whole-body sweat sodium concentration at 40 min differed significantly from all other time points (all *p* < 0.05). No significant differences were observed between the 130-, 220-, and 290 min time points (all *p* > 0.20). Mean WBSSC were 48.0 ± 14.6 mmol·L^−1^ and 47.6 ± 13.9 mmol·L^−1^ for P and A_L_, respectively.

Table 3 presents the within-trial temporal changes in WBSSC. The mean difference, typical variation, and typical variation expressed as a coefficient of variation were relatively similar between trials at each time point. The typical variation in WBSSC across all consecutive time points within each trial was ≤7 mmol·L^−1^. The typical variation in WBSSC and the associated coefficient of variation over the 5 h exercise period, defined as the cluster of the four time points within each trial, were 7.2 mmol·L^−1^ and 16.5% for P, and 6.1 mmol·L^−1^ and 13.9% for A_L_, respectively.

Table 4 summarizes the between-trial reproducibility in WBSSC. The mean difference, typical variation, and typical variation expressed as a coefficient of variation were generally similar across consecutive time points. The typical variation in WBSSC between trials for consecutive time points was ≤7 mmol·L^−1^. Over the 5 h exercise period, the typical variation in WBSSC and the associated coefficient of variation between trials were 5.6 mmol·L^−1^ and 12.7%, respectively. Intraclass correlation coefficients were all above 0.80.

### 3.5. Prediction of Whole-Body Sweat Sodium Concentration During Exercise

**Figure 2:**
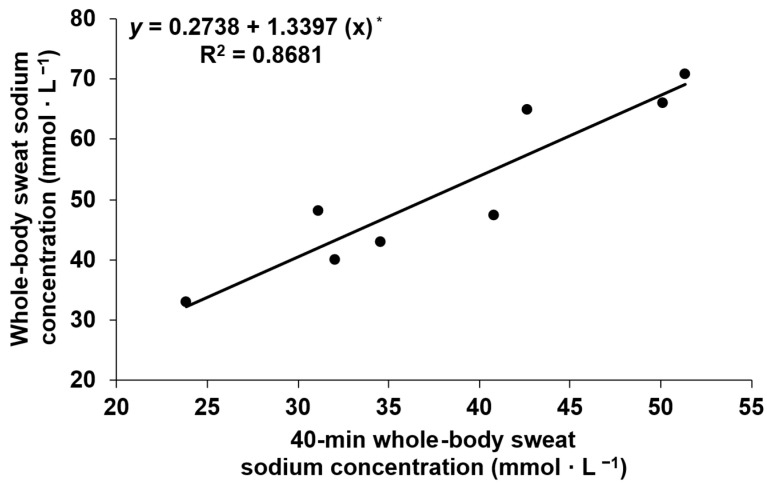
Relationships between the 40 min whole-body sweat sodium concentration values with the time-weighted average whole-body sweat sodium concentration values measured during exercise. * = *p* < 0.05.

Given the minimal physiologically relevant variation in WBSSC observed between trials, data from both trials were pooled for the analysis. Figure 2 presents the regression analyses relating the WBSSC at the 40 min time point to the time-weighted average WBSSC observed throughout the exercise period. The relationship between variables was significant and strong, with an *R*^2^ value of 0.87. The predictive capacity of the model was useful with a standard error of the estimate (SEE) of 5.4 mmol·L^−1^. The 95% confidence interval around the slope coefficient was 0.82 to 1.86 mmol·L^−1^. The bootstrap mean slope (1.340) closely matched the original estimate (1.3397), with a bootstrap SD of 0.190 and a 95% bootstrap confidence interval of 0.93–1.70, indicating moderate parameter stability (0.190/1.3397). Influence diagnostics revealed no influential observations (all Cook’s distances < 0.20), supporting robustness of the regression equation.

## 4. Discussion

Sodium losses through sweating can be estimated as the product of whole-body sweat rate and WBSSC [26]. Although the importance of replacing sodium losses during prolonged exercise is widely acknowledged [3,4,5], practical guidance on how to implement sodium replacement remains limited, particularly with respect to the integration of WBSSC into the sodium loss calculations. Specifically, the extent to which WBSSC varies within and between prolonged exercise sessions, the feasibility of predicting WBSSC during exercise, and the potential influence of hydration strategies on WBSSC remain incompletely characterized. The present study addressed these gaps and demonstrated that, within the context of the current study and small sample size: (1) WBSSC exhibits only trivial variation within and between prolonged cycling exercise sessions; (2) WBSSC during prolonged cycling can reasonably be predicted using a single sweat sample; and (3) the hydration strategy (P vs. A_L_ drinking) does not meaningfully influence WBSSC.

Within both trials, changes in WBSSC were small. Indeed, typical variations in WBSSC of between 2.8 mmol·L^−1^ and 7.2 mmol·L^−1^ (64 mg·L^−1^ to 166 mg·L^−1^) were observed across consecutive time points in each trial. Furthermore, over the entire exercise duration in each trial, the typical variation in WBSSC varied between 6.1 mmol·L^−1^ to 7.2 mmol·L^−1^. However, it must be emphasized that these variations in WBSSC are based on a predictive model of WBSSC, and not on a direct WBSSC measurement. The present results contrast with those of Montain et al. [12], who reported changes in WBSSC during 7 h of exercise ≤ 2 mmol·L ^−1^. However, comparisons with their results are difficult given that their exercise protocol was different than ours and the procedure they used to collect sweat samples may have been associated with hidromeiosis, thereby invalidating their findings [1]. In the present study, only the 40 min WBSSC differed significantly from later values, suggesting that WBSSC had stabilized after this period. This observation is consistent with the expectation that early exercise sweat samples obtained before steady-state maximal sweat production is achieved for a given exercise intensity should be associated with lowered WBSSC [1]. Although no sweat samples were collected between 40 and 130 min, a time-weighted average WBSSC across this interval closely matched the values measured later in exercise, supporting the likelihood that WBSSC stabilized soon after the first 40 min of exercise. Notwithstanding statistical significance, the changes in WBSSC observed over time between time points in each trial can be considered physiologically relatively trivial. Indeed, it matched the between trials typical variation in WBSSC observed in the current study (discussed later). Collectively, these findings indicate that once a steady whole-body sweat rate is established, physiologically meaningful changes in WBSSC during 5 h of fixed-intensity cycling exercise are negligible. From a practical standpoint, the present findings imply that, assuming whole-body sweat rate remains relatively stable, an even distribution of sodium replacement throughout prolonged exercise is appropriate.

The typical variation in WBSSC between trials was comparable to that observed within trials, and no systematic between-trial differences were detected. Baker et al. [19] reported a WBSSC coefficient of variation of approximately 16% during a 90 min exercise bout in the heat. The between-trial coefficient of variation of ~13% observed in the present study corroborates these findings while extending them to prolonged cycling exercise performed by highly trained endurance athletes. This observation has several important implications. First, it indicates that the hydration strategy, whether P or A_L_, does not meaningfully influence WBSSC when dehydration is maintained below moderate levels. This is consistent with previous work showing no effect of fluid intake on WBSSC when body mass losses remain below ~2.5% [9]. Second, the trivial between-trial variability in WBSSC supports the use of a single WBSSC value to inform sodium replacement strategies across comparable prolonged exercise sessions. Third, it suggests that WBSSC fluctuations of approximately ±13% between exercise or sweat testing sessions should be considered physiologically non-meaningful. Finally, these findings imply that WBSSC values obtained during a sweat composition test may be transferable to other prolonged exercise sessions performed under similar conditions.

A key finding of this study is that WBSSC during prolonged exercise can be fairly predicted from a regression equation using a single sweat sample collected within the first 40 min of exercise. Indeed, the coefficient of determination associated with the equation was strong (87%), implying that there was a robust association between the 40 min WBSSC and the time-weighted average WBSSC. The SEE represents the sample-based estimate of the population residual standard deviation and reflects the expected magnitude of prediction error of a regression model. Interestingly, the predictive error of the model was low (5.4 mmol·L^−1^) and inconsequential from a physiological standpoint, indicating that the model-based prediction is accurate enough to be useful. Nevertheless, the stability of the slope coefficient was only moderate, indicating that further studies with a greater number of participants are needed to improve the calibration of the model. A potential limitation of our observation is that sweat collected at min 40 was compared with WBSSC values obtained later within the same exercise session. It remains unclear whether the predictive capacity of the 40 min sweat sample would be preserved for a prolonged exercise bout that does not immediately follow sweat collection, as is the case for conventional sweat-testing protocols which are conducted independently of, and not directly before, a bout of prolonged exercise. Nonetheless, the absence of differences between the WBSSC measured both at 40 min and later during exercise across trials suggests that this may be achievable. Accordingly, the primary practical implication of these findings is that WBSSC can be accurately predicted during prolonged cycling exercise from a single sweat sample collected over 20 min following 20 min of exercise. Although the 40 min sweat sample could potentially serve as a proxy for the WBSSC expected during exercise, the use of the regression equation is likely to improve estimation precision and, consequently, the accuracy of sodium supplementation strategies during exercise. We emphasize that the predictive capacity of our model should be framed as hypothesis-generating, since validity across separate exercise sessions or field testing remains unverified.

Our findings should be interpreted in light of several limitations. First, the sample size was modest, primarily due to the demanding nature of the exercise protocol, which limited the feasibility of participant recruitment. Nevertheless, the primary outcomes focused on within-subjects variability and reproducibility, which are less dependent on large sample sizes than between-group comparisons. Moreover, the statistical power for the comparison analyses was high, and our regression model showed good predictive power and moderate stability. Second, heat acclimatization status can influence WBSSC [1]; however, participants were not heat-acclimatized at the time of testing. Third, the WBSSC may also vary with anatomical sampling site, analytical method, collection technique, sampling duration, and collection timing within exercise [1]. Consequently, the present findings apply specifically to forearm sweat samples collected using absorbent patches during 20 min of exercise and analyzed via the ion-selective electrode technique. Fourth, these findings apply specifically to prolonged exercise formats (5–6 h in duration) characterized by a constant exercise intensity. It is premature to suggest that these findings can be generalized to ultra-endurance exercise; nevertheless, they provide a reasonable basis for decision-making. Fifth, it is unclear whether a sweat sample collected between minute 20 and minute 40 of an exercise bout performed at a different intensity or under environmental conditions distinct from those employed in the present study would accurately predict the mean WBSSC observed during prolonged exercise using the proposed regression equation. Sixth, regional sweat sodium concentration was converted to WBSSC using an equation validated during a 90 min exercise protocol. However, there is currently no evidence indicating that the relationship between regional and WBSSC remains constant or linear during ultra-endurance exercise. Finally, these results are limited to highly trained male endurance athletes performing prolonged cycling exercise in the heat under conditions of moderate humidity.

## 5. Conclusions

Our results indicate that, during prolonged cycling exercise performed by trained male endurance athletes, WBSSC: (1) varies trivially within and between exercise sessions; (2) is not meaningfully influenced by P or A_L_ fluid intake when dehydration remains below moderate levels and; (3) can be predicted with reasonable accuracy using a single sweat sample collected between min 20 and 40 following the onset of exercise. These conclusions should be interpreted within an important limitation of this study in mind, which is related to the small sample size. Hence, further studies with more participants are required to improve the precision of the current observations. Nevertheless, these findings provide practical insight for athletes and practitioners seeking to implement evidence-based sodium replacement strategies during prolonged cycling exercise.

## 6. Perspectives

Because estimation of sodium losses through sweating requires assessment of WBSSC in addition to whole-body sweat rate, the findings of the present study have practical implications for sodium replacement recommendations during prolonged exercise. Specifically, our results suggest that during prolonged cycling performed at comparable intensities and under similar environmental conditions, sodium replacement (1) may be distributed evenly over the course of exercise; (2) can be applied consistently across exercise sessions; (3) can be predicted with acceptable accuracy from a single sweat sample collected for 20 min, 20 min following the onset of exercise; and (4) is not meaningfully influenced by the manner in which fluid is consumed, i.e., P vs. A_L_. Importantly, these recommendations must be considered in light of the limitations associated with the study.

## Figures and Tables

**Table 1:** Baseline characteristics of the participants.

Characteristics	Mean ± SD
Age (y)	26 ± 6
Height (cm)	178 ± 7
Body mass (kg)	71 ± 6
BSA (m^2^)	1.9 ± 0.1
Fat mass (%)	8 ± 2
Lean body mass (%)	92 ± 2
Peak oxygen consumption (mL·kg^−1^·min^−1^)	67 ± 4
State of heat acclimatization *	Unacclimatised

BSA = body surface area. * = participants were tested outside the summer months.

**Table 2:** Hydration state before the experimental trials.

Parameters	Programmed Drinking	*Ad Libitum* Drinking	*p* Value
Body mass (kg)	71.6 ± 5.8	70.8 ± 5.5	0.16
Urine specific gravity	1.014 ± 0.011	1.012 ± 0.009	0.50
Whole blood osmolality (mOsmol·kg^−1^)	301.9 ± 11.0	300.6 ± 8.6	0.64
Thirst perception (AU)	4 ± 1	4 ± 1	1.00

Results are means ± SD. AU = arbitrary units.

**Table 3:** Within-trials changes in whole-body sweat sodium concentration.

**Variables**	**Programmed Drinking**			
	**Time Points (min)**			
	**130 vs. 40**	**220 vs.** **130**	**290 vs.** **220**	**290 vs.** **40**
Mean difference (mmol·L^−1^) (± SD)	13.2 ± 9.9	3.2 ± 5.9	−4.1 ± 4.1	12.5 ± 10.1
*T*-test *p* value	0.01	0.20	0.03	0.01
Typical variation (mmol·L^−1^)	±7.0	±4.1	±2.9	±7.2
Typical variation as a coefficient of variation (%)	±15.6	±8.0	±5.7	±16.1
**Variables**	***Ad Libitum*** **Drinking**			
	**Time Points (min)**			
	**130 vs. 40**	**220 vs.** **130**	**290 vs.** **220**	**290 vs.** **40**
Mean difference (mmol·L^−1^) (± SD)	10.5 ± 4.9	2.1 ± 4.0	0.4 ± 6.6	14.4 ± 10.2
*T*-test *p* value	0.01	0.15	0.86	<0.01
Typical variation (mmol·L^−1^)	±3.5	±2.8	±4.7	±7.2
Typical variation as a coefficient of variation (%)	±8.2	±5.6	±8.9	±15.8

**Table 4:** Between-trials changes in whole-body sweat sodium concentration.

Variables	Time Points (min)			
	40	130	220	290
Mean difference (mmol·L^−1^) (± SD)	0.1 ± 5.4	2.7 ± 8.9	1.1 ± 4.2	−1.8 ± 9.4
*T*-test *p* value	0.95	0.36	0.53	0.61
Typical variation (mmol·L^−1^)	±3.8	±6.2	±2.9	±6.6
Typical variation as a coefficient of variation (%)	±10.6	±13.5	±6.0	±13.7
Intraclass correlation coefficient (ICC)	0.869 *	0.810 *	0.968 *	0.808 *

* = *p* < 0.05.

## Data Availability

The data will be made available from the corresponding author upon reasonable request.
